# General Therapeutics and Materia Medica; Adapted for a Medical Text Book

**Published:** 1850-09

**Authors:** 


*General Therapeutics and Materia Medica; adapted for a Medical Text Book.* By Robley Dunglison, M. D., Professor of Institutes of Medicine in Jefferson Medical College, &c. &c. *One hundred and eighty-two illustrations. Fourth edition, revised and improved, in two volumes.* Philadelphia. Lea & Blanchard.

Good wine, it is said, needs no bush. The wide-spread reputation and rapid sale of Dr. Dunglison's works are the best exponents of their acceptableness to the practitioner and student, for the latter of whom they are more especially intended. In announcing a fourth edition, we are informed that the work has been subjected to a thorough revision, both as regards the style and matter. The remedial agents of recent introduction have been incorporated in their appropriate places; the number of illustrations has been greatly increased; and a copious index of diseases and remedies has been appended, which can scarcely fail to add to the value of the work to the therapeutical inquirer. The well known habits of industry of the author are a sufficient guaranty for the faithfulness and completeness with which the work has been executed. We commend it most heartily to both practitioner and student, assured that they will find in it a mine of treasure.

